# Effect of a patient education and rehabilitation program on anxiety, depression and quality of life in muscle invasive bladder cancer patients treated with adjuvant chemotherapy

**DOI:** 10.1097/MD.0000000000017437

**Published:** 2019-11-01

**Authors:** Zhonghui Li, Dan Wei, Chenxi Zhu, Qing Zhang

**Affiliations:** a Department of Urology Surgery; b Department of Thoracic Surgery, The Central Hospital of Wuhan, Tongji Medical College, Huazhong University of Science and Technology, Wuhan 430014, China.

**Keywords:** adjuvant chemotherapy, anxiety and depression, muscle invasive bladder cancer, patient education and rehabilitation program, quality of life

## Abstract

This study aimed to investigate the effect of a patient education and rehabilitation program (PERP) on anxiety, depression, and quality of life in muscle invasive bladder cancer (MIBC) patients underwent adjuvant chemotherapy.

One hundred and thirty MIBC patients about to receive adjuvant chemotherapy with 4-cycle gemcitabine and cisplatin (GC) regimen (16 weeks) were consecutively enrolled and randomly allocated into PERP group and control group as 1:1 ratio. Hospital Anxiety and Depression Scale (HADS) anxiety and depression scores and Quality of Life Questionnaire (QLQ-C30) scores were assessed before treatment (W0) and after treatment (W16).

After 16-week treatment, PERP group exhibited decreased HADS anxiety score (*P* = .036), ΔHADS anxiety score (W16-W0) (*P* < .001) and percentage of anxiety patients (*P* = .019) compared to control group. And PERP group presented with numerically reduced HADS depression score but without statistical significance (*P* = .076) compared to control group, while lower ΔHADS depression score (W16-W0) (*P* = .014) and percentage of depression patients (*P* = .015) compared to control group. As to quality of life, QLQ-C30 global health status score (*P* = .032), Δglobal health status score (W16-W0) (*P* = .003) and Δfunctional score (W16-W0) (*P* = .005) were higher in PERP group compared to control group. However, no difference of QLQ-C30 functional score (*P* = .103), QLQ-C30 symptom score (*P* = .808) or Δsymptom score (W16-W0) (*P* = .680) was observed between two groups.

PERP relieves anxiety, depression and improves quality of life in MIBC patients underwent adjuvant chemotherapy.

## 1 Introduction

Bladder cancer is one of the top 10 commonly diagnosed malignancies in China. According to the 2015 cancer statistics, there are 8,05,000 new cases and 3,29,000 deaths by it annually in China, whose number is still increasing with time.^[1]^ Muscle invasive bladder cancer (MIBC), accounting for 30% of all bladder cancer, is attracting great attention due to high metastatic rate and mortality.^[2]^ The current treatment for MIBC is cystectomy with pelvic lymph node dissection (if there is lymph node metastasis), and adjuvant chemotherapy is used to consolidate the treatment and prevent recurrence.^[3,4]^ However, adjuvant chemotherapy often causes discomfort in patients that promotes the generation of anxiety and depression and reduces the quality of life of patients^[5,6]^ In addition, mood disorders such as depression and cognitive impairment are reported to be strong contributors to suicide intension.^[7]^ Therefore, it of great need to actively investigate in approaches that relief anxiety and depression in MIBC patients undergoing adjuvant chemotherapy.

Accumulating literature has shown that rehabilitation is not only able to improve physical function but also to relieve psychological depression of cancer survivors.^[8]^ And several education and rehabilitation programs are shown to greatly reduce the anxiety and depression of cancer patients after surgery or underwent chemotherapies.^[9–11]^ According to the previous evidence, we hypothesized that education and rehabilitation program could also relieve psychological distress and improve the quality of life in MIBC patients. Therefore, this study designed a comprehensive patient education and rehabilitation program (PERP), which included education session, psychological nursing, individual exercise rehabilitation guidance and communication activity, and investigated the efficacy of PERP on reducing anxiety and depression, improving quality of life in MIBC patients undergoing adjuvant chemotherapy.

## 2 Materials and methods

### 2.1 Patients

In this randomized, controlled study, a total of 130 MIBC patients about to receive GC (gemcitabine + cisplatin) adjuvant therapy at The Central Hospital of Wuhan from January 2015 to December 2017. The inclusion criteria were as follows: first, diagnosed as MIBC confirmed by the clinicopathologic examinations; second, intended to underwent GC adjuvant therapy after surgery; third, age above 18 years; fourth, life expectancy more than 12 months; fifth, able to understand the study contents and fulfill the questionnaires independently; and sixth, able to regularly followed up. The exclusion criteria were: first, concomitant with other malignancies or tumors; second, severe cognitive impairment or schizophrenia; third, contraindications to GC chemotherapy; fourth, neoadjuvant therapy; and fifth, pregnant or lactating woman. This study was approved by the Ethics Committee of The Central Hospital of Wuhan (Ethics Committee approval number: 2014-8), and all patients or their guardians signed the informed consents before initiation of study.

### 2.2 Baseline data collection

After enrollment, patients’ baseline characteristics were documented, mainly including age, gender, body mass index (BMI), highest education, smoke, drink, hypertension, hyperlipidemia, diabetes, Eastern Cooperative Oncology Group (ECOG) score, tumor stage, and pathological differentiation.

### 2.3 Randomization

Random grouping sequence was developed by an independent analyzer using the block randomization method with block size of 4 on the SAS version 9.0 software (SAS Institute, Inc., Cary, North Carolina). A nurse without involvement in other parts of study was responsible for the assignment of patients according to the grouping sequence as 1:1 ratio. Consequently, 65 patients were randomly assigned to the PERP group, and 65 patients were randomly allocated to the control group.

### 2.4 Adjuvant chemotherapy

After surgery, conventional care was given to all patients, and the GC regimen was administered within one month, as follows: gemcitabine 1000 mg/m^2^ at day 1, 8, and 15, plus cisplatin 70 mg/m^2^ at day 2 every 28 days for 4 cycles.

### 2.5 Interventions

Patients in both two groups were given education materials for rehabilitation, including contents of basic disease knowledge, treatment process, matters need attention, self-management and surveillance, diet care, psychological care, exercise rehabilitation, and so on. And a systematic guidance was delivered to all patients before discharge from hospitalization. On the basis of conventional care, PERP was performed for patients in the PERP group, which was lasted for 16 weeks and delivered every two weeks by trained nurses in the rehabilitation center of the hospital. The PERP consisted of four parts: education sessions, psychological nursing, exercise rehabilitation guidance, and communication activities, and the details were as follows: first, education session was given to the patients, and each session was lasted for 30 minutes, during which, trained nurses explained the contents of the education materials for patients in detail; second, psychological nursing was carried out in a duration of 30 minutes at each time, during which, nurses actively communicated with patients and their families to establish a good medical relationship, as far as possible to help patients solve problems, eliminate fear, anxiety and even despair, to encourage patients to face the disease with a positive attitude to actively cooperate with treatment; third, individual exercise rehabilitation guidance was given to patients according to the patients’ rehabilitation status. Each guidance lasted for 30 minutes, and the individual exercise program was developed by communicating with patients and their families. The implementation of the exercise program was required to be documented by patients or their families, which was checked by the trained nurses every two weeks for surveillance; and fourth, communication activity was conducted when each education session was complete, during which, trained nurses encouraged patients and their families to communicate with other patients, and organized patients to join some recreational activities (such as chess, chorus, calligraphy, literary and artistic performances, etc). For the patients in the control group, usual rehabilitation recommendations were given by their treating physician when they underwent adjuvant chemotherapy.

### 2.6 Assessment

Anxiety, depression, and quality of life of all patients were assessed at baseline (M0) and after intervention (W16), using the Mandarin versions of Hospital Anxiety and Depression Scale (HADS) and the European Organization for Research and Treatment of Cancer Quality of Life Questionnaire (QLQ-C30 Scale). Before initiation of study, all patients were given instructions that explained how to fulfill those scales, then an independent nurse (unaware of assignment of patients) was responsible for collecting the scales (at W0 and W16) and calculating the HADS-anxiety score, HADS-depression score, QLQ-C30 global health status score, QLQ-C30 functional score, and QLQ-C30 symptom score, correspondingly. According to the HADS-anxiety score and HADS-depression score, the severity of anxiety and depression was assessed as follows: 0–7, no anxiety/depression; 8–10, mild anxiety/depression; 11–14, moderate anxiety/depression; 15–21, severe anxiety/depression.^[12]^ According to QLQ-C30 Scoring Manual, all scales and single-item measures were ranged score of 0–100, higher scores in the global health status scale and functional scale indicated better health state and function, while higher score in the symptoms scale indicated worse symptoms.^[13]^

### 2.7 Statistical analysis

The calculation of sample size was based on a significance level of 0.05% and 15% attrition rate, and the current sample size ensured a power more than 80%. Statistical analysis was performed by use of SPSS version 22.0 (IBM, Chicago, IL). Count data were expressed as count (percentage), and continuous data were described as mean and standard deviation (SD). Comparison between two groups was determined by the Chi-square test, Wilcoxon rank sum test or Student's *t* test. *P* value < .05 was considered statistically significant.

## 3 Results

### 3.1 Study flow

**Figure 1:**
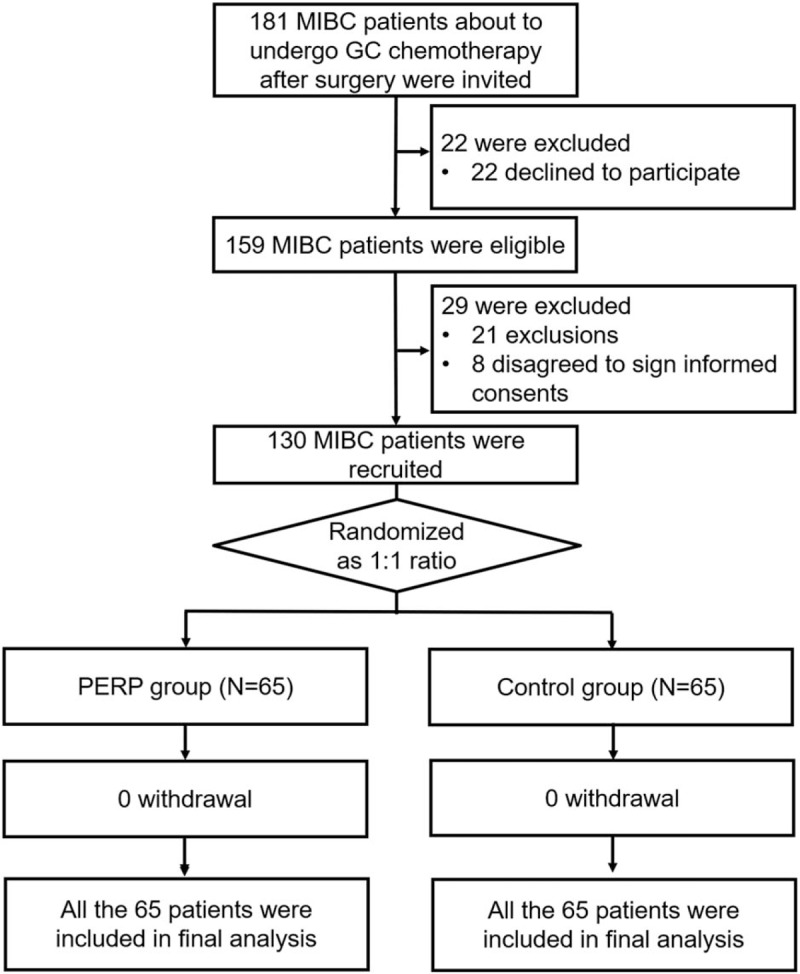
Study flow. GC = gemcitabine + cisplatin, MIBC = muscle invasive bladder cancer, PERP = patient education and rehabilitation program.

A total of 181 MIBC patients who were about to undergo GC chemotherapy after surgery were invited, while 22 of them were excluded because they declined to participate (Fig. 1). The remaining 159 MIBC patients were eligible while 29 of them were excluded including 21 patients meeting the exclusion criteria and 8 patients disagreed to sign the informed consents. There were 130 MIBC patients who were recruited and were randomized as 1:1 ratio into PERP group (N = 65) and control group (N = 65). No patient withdrew in PERP group or control group, and all 65 patients in PERP group were included in final analysis and all 65 patients in control group were included in final analysis.

### 3.2 Baseline characteristics of MIBC patients

MIBC patients in PERP group were with mean age of 57.5 ± 14.0 years, and 49/16 of them were males/females (Table 1). Patients in control group were with mean age of 57.9 ± 13.2 years, and 50/15 of them were males/females. There was no difference in age (*P* = .867), gender (*P* = .837), BMI (*P* = .623), highest education (*P* = .258), history of smoke (*P* = .859), drink (*P* = .458), hypertension (*P* = .375), hyperlipidemia (*P* = .357), diabetes (*P* = .115), ECOG score (*P* = .983), tumor stage (*P* = .118), or pathological differentiation (*P* = .276) between PERP and control groups. The detailed characteristics of MIBC patients between two groups were listed in Table 1.

**Table 1:**
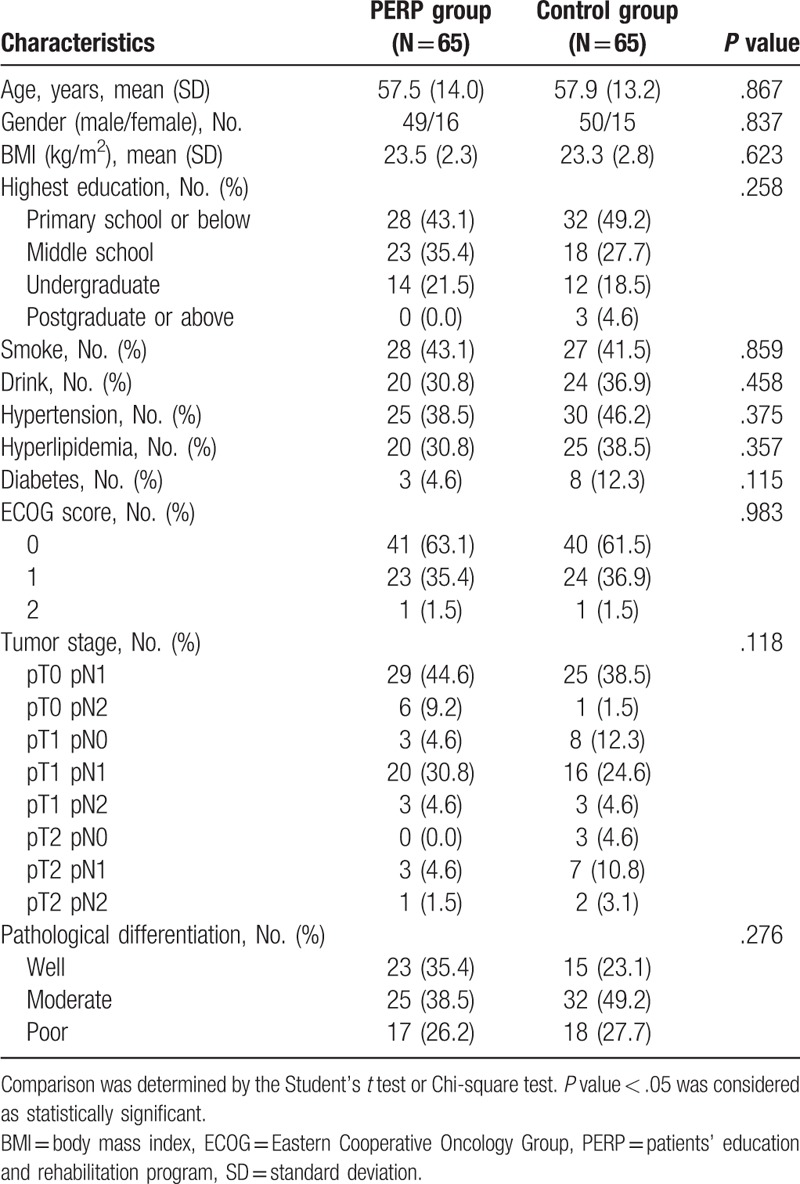
Baseline characteristics of patients.

### 3.3 Comparison of anxiety between PERP group and control group

The baseline HADS anxiety score (*P* = .768), proportion of anxiety patients (*P* = .850), and anxiety severity (*P* = 1.000) were similar between PERP group and control group (Table 2). At 16 weeks after intervention, HADS anxiety score (*P* = .036), ΔHADS-anxiety score (W16-W0) (*P* < .001), and proportion of patients with anxiety (*P* = .019) was lower in PERP group compared with control group. These data implied that PERP reduced anxiety in MIBC patients undergoing adjuvant chemotherapy.

**Table 2:**
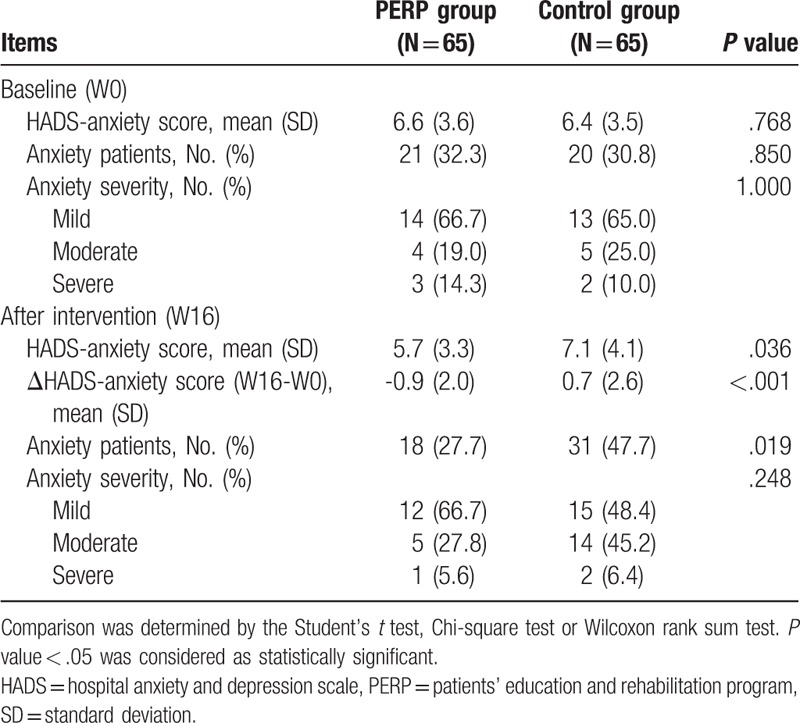
Comparison of HADS-anxiety between two groups.

### 3.4 Comparison of depression between PERP group and control group

There was no difference in baseline HADS-depression score (*P* = .955), proportion of depression patients (*P* = .824) or depression severity (*P* = .894) between PERP group and control group (Table 3). And at 16 weeks after intervention, HADS-depression score was numerically lower in PERP group compared with control group but without statistical significance (*P* = .076); ΔHADS-anxiety score (W16-W0) (*P* = .014) and proportion of patients with depression (*P* = .015) were smaller in PERP group compared with control group. The above evidence suggested that PERP reduced depression in MIBC patients undergoing adjuvant chemotherapy to some extent.

**Table 3:**
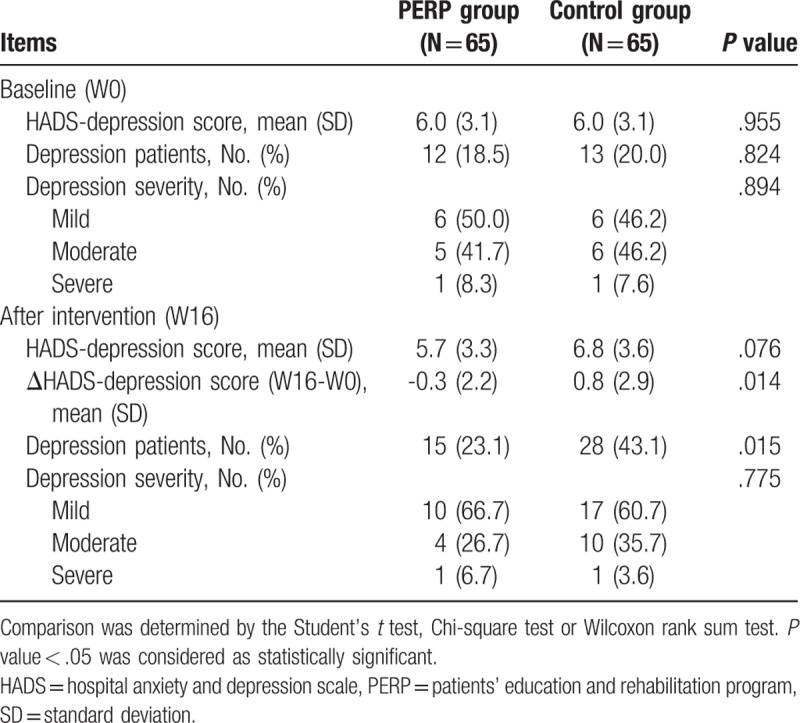
Comparison of HADS-depression between two groups.

### 3.5 Comparison of quality of life between PERP group and control group

The baseline QLQ-C30 global health status score (*P* = .452), QLQ-C30 functional score (*P* = .763), and QLQ-C30 symptom score (*P* = .873) were similar between PERP group and control group (Table 4). At 16 weeks after intervention, there were improvement in QLQ-C30 global health status score in both groups. In PERP group, QLQ-C30 global health status score (*P* = .032) and Δglobal health status score (W16-W0) (*P* = .003) were greater compared with control group. Besides, QLQ-C30 functional score was increased in both groups after 16 weeks. In PERP group, QLQ-C30 functional score was higher but with no statistical significance (*P* = .103) and Δfunctional score (W16-W0) (*P* = .005) was higher compared with control group. As for QLQ-C30 symptom score, no difference of QLQ-C30 symptom score (*P* = .808) or ΔQLQ-C30 symptom score (W16-W0) (*P* = .680) was observed between PERP group and control group.

**Table 4:**
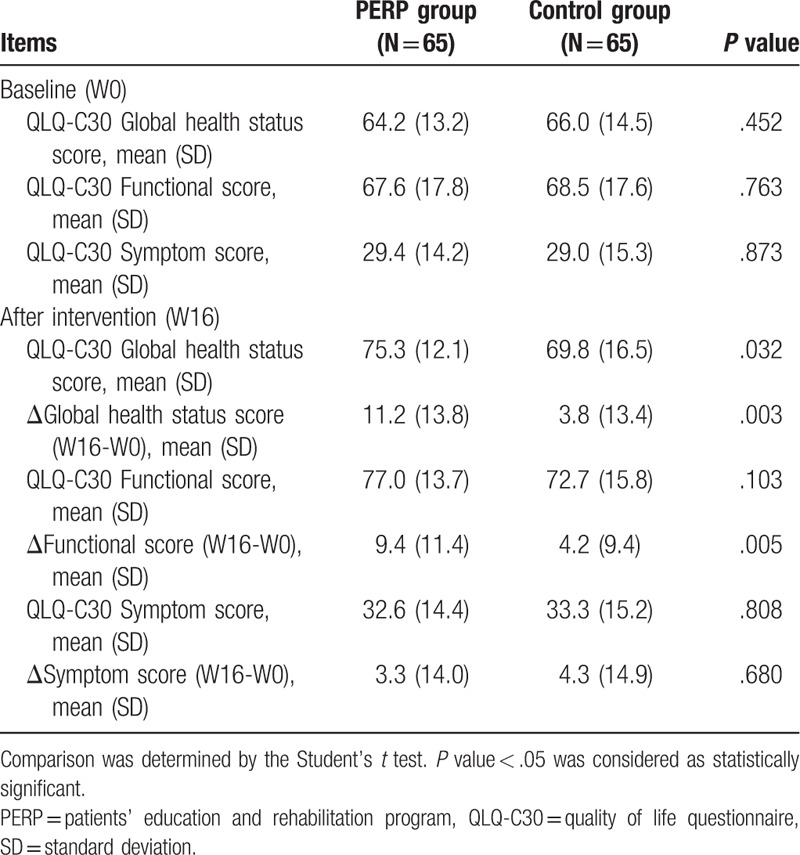
Comparison of QLQ-C30 between two groups.

## 4 Discussion

Our study showed that in MIBC patients receiving adjuvant chemotherapy, first, the anxiety and depression levels in the PERP group were lower than those in the control group and second, the improvement of QLQ-C30 global health status score and QLQ-C30 functional score in PERP group was higher than that in control group.

Hospital Anxiety and Depression Scale (HADS), SDS/SAS Zung Scale, and Hamilton scale are widely used assessments for emotional symptoms in various medical conditions.^[14,15]^ However, Hamilton anxiety scale is a psychological questionnaire used by clinicians with specific certification, and SDS/SAS Zung Scale contains a complicated scoring system that needs conversion of the score. Therefore, HADS, which is a relatively simple and accessible assessment tool was adopted in this study. In addition, the European Organization for Research and Treatment of Cancer Quality of Life Questionnaire (QLQ-C30 Scale) was used for assessing quality of life. The reason was that this was a multidimensional instrument for quality of life measurement specifically in cancer patients, and has been widely applied by various clinical trials in cancer field.^[16]^

A previous meta-analysis reveals that mental and cognitive intervention is especially effective in reducing depression of cancer patients, and long-term observation shows that the 15-year incidence of depression is lower and quality of life is better in intervention group compared with control group.^[8]^ Another internet-based psychological rehabilitation program exhibits that the anxiety and depression levels of early breast cancer patients who participated in the psychological intervention were improved, and their cooperation in the project were very high.^[17]^ These studies suggest that education and rehabilitation programs improve anxiety and depression in cancer patients. However, the effect of such program on anxiety and depression in MIBC patients undergoing adjuvant chemotherapy is rarely reported. Our study designed a comprehensive program PERP, and explored its efficacy in reducing anxiety and depression in MIBC patients receiving adjuvant chemotherapy. We observed that anxiety and depression were relieved in PERP group compared with control group after intervention, which indicated that PERP effectively reduced anxiety and depression in MIBC patients undergoing adjuvant chemotherapy. Here are the possible explanations: first, patients in PERP group are more educated with the disease and treatment, and are more capable of self-managing emotion. Therefore, they have higher acceptance to disease and treatment-related side effects, lower anxiety and depression than the control group and second, patients in the PREP group received psychological counseling and frequent communication with medical staff, which directly and indirectly alleviated mental stress. Therefore, compared with patients in the control group, patients in the PREP group had lower levels of anxiety and depression.

Cancer and its treatment not only causes anxiety and depression, but also have a huge impact on patients’ life, which also makes the quality of life of cancer survivors become another important indicator of cancer management and is included in the evaluation for the efficacy of some postoperative rehabilitation nursing programs.^[18,19]^ For instance, a four-week, multi-channel nursing project involving psychological intervention and physical rehabilitation alleviates the cancer-related symptoms of breast cancer patients, such as fatigue, pain, nausea, and postoperative muscle strength, and improves the quality of life.^[9]^ Another program that involved both psychological and physical rehabilitation activities showed that cancer patients who received 12 weeks of rehabilitation care had higher quality of life indicators than the control group.^[11]^ In addition, an education program for patients with esophageal cancer also shows that active patient education and doctor-patient communication can significantly improve the quality of life.^[10]^ These comprehensive rehabilitation programs among different cancer patients all suggest that these programs can improve the quality of life for cancer patients. However, limited study investigates the effect of comprehensive rehabilitation program on quality of life in MIBC patients receiving adjuvant chemotherapy. In the present study, PERP increased the QLQ-C30 global health status score and QLQ-C30 functional score, which implied that PERP effectively improved the quality of life in MIBC patients undergoing adjuvant chemotherapy. This can be due to that: first, the patient-specific exercise rehabilitation in PREP may directly improve patients’ physical health, such as physical function, and improve their quality of life and second, PREP reduces anxiety and depression in MIBC patients, and the improvement of mental state is likely to improve the health status of patients, thus contributing to the improvement of patients’ quality of life.

This study was the first to evaluate the effect of PERP on the anxiety, depression and quality of life in MIBC patients receiving adjuvant chemotherapy, which would provide certain evidence for the treatment and management of MIBC patients clinically. However, there were still some shortcomings of this study. Firstly, the sample size of this study was slightly higher than the calculated minimum sample size, so it was necessary to further verify the results in a larger sample size in the future. Secondly, considering the cost of randomized controlled studies and the difficulties in long-term management of patients, this study did not evaluate the effect of PERP on patients’ long-term anxiety, depression and quality of life, which needed further exploration. Additionally, the long-term effect of PERP on survival was not evaluated in this study, which needed further evaluation as well. Finally, this was a single-centered study, and the patients were mainly from central China. Therefore, a cross-regional and multi-center study will be necessary to verify the results of this study.

## 5 Conclusion

In conclusion, PERP relieves the anxiety, depression and improves quality of life of MIBC patients receiving adjuvant chemotherapy, which implies that education and rehabilitation programs are beneficial in post-operative management of MIBC regarding mental as well as general well-being.

## Author contributions

**Conceptualization:** Dan Wei, Qing Zhang.

**Data curation:** Zhonghui Li, Dan Wei, Qing Zhang.

**Formal analysis:** Zhonghui Li, Dan Wei, Qing Zhang.

**Funding acquisition:** Zhonghui Li, Dan Wei, Chenxi Zhu.

**Investigation:** Zhonghui Li, Dan Wei, Chenxi Zhu, Qing Zhang.

**Methodology:** Zhonghui Li, Dan Wei, Qing Zhang.

**Project administration:** Zhonghui Li.

**Resources:** Zhonghui Li, Chenxi Zhu.

**Software:** Dan Wei, Chenxi Zhu.

**Supervision:** Dan Wei, Chenxi Zhu, Qing Zhang.

**Validation:** Zhonghui Li, Dan Wei, Chenxi Zhu, Qing Zhang.

**Visualization:** Zhonghui Li, Dan Wei, Chenxi Zhu, Qing Zhang.

**Writing – original draft:** Zhonghui Li, Dan Wei, Chenxi Zhu, Qing Zhang.

**Writing – review & editing:** Zhonghui Li, Dan Wei, Chenxi Zhu, Qing Zhang.

## References

[1] Chen W Zheng R Baade PD Cancer statistics in China, 2015. CA Cancer J Clin 2016;66:115–32. 26808342 10.3322/caac.21338

[2] Kamat AM Hahn NM Efstathiou JA Bladder cancer. Lancet 2016;388:2796–810. 27345655 10.1016/S0140-6736(16)30512-8

[3] Meeks JJ Bellmunt J Bochner BH A systematic review of neoadjuvant and adjuvant chemotherapy for muscle-invasive bladder cancer. Eur Urol 2012;62:523–33. 22677572 10.1016/j.eururo.2012.05.048

[4] Shimizu F Muto S Taguri M Effectiveness of platinum-based adjuvant chemotherapy for muscle-invasive bladder cancer: a weighted propensity score analysis. Int J Urol 2017;24:367–72. 28281310 10.1111/iju.13324

[5] Mohamed NE Gilbert F Lee CT Pursuing quality in the application of bladder cancer quality of life research. Bladder Cancer 2016;2:139–49. 27376136 10.3233/BLC-160051 PMC4927895

[6] Vartolomei L Ferro M Mirone V Systematic review: depression and anxiety prevalence in bladder cancer patients. Bladder Cancer 2018;4:319–26. 30112443 10.3233/BLC-180181 PMC6087432

[7] Pompili M Venturini P Lamis DA Suicide in stroke survivors: epidemiology and prevention. Drugs Aging 2015;32:21–9. 25491561 10.1007/s40266-014-0233-x

[8] Akechi T Okuyama T Onishi J Psychotherapy for depression among incurable cancer patients. Cochrane Database Syst Rev 2008;CD005537. 10.1002/14651858.CD005537.pub2 PMC6464138 18425922

[9] Do J Cho Y Jeon J Effects of a 4-week multimodal rehabilitation program on quality of life, cardiopulmonary function, and fatigue in breast cancer patients. J Breast Cancer 2015;18:87–96. 25834616 10.4048/jbc.2015.18.1.87 PMC4381129

[10] Pool MK Nadrian H Pasha N Effects of a self-care education program on quality of life after surgery in patients with esophageal cancer. Gastroenterol Nurs 2012;35:332–40. 23018169 10.1097/SGA.0b013e3182605f86

[11] Lee YH Lai GM Lee DC Promoting physical and psychological rehabilitation activities and evaluating potential links among cancer-related fatigue, fear of recurrence, quality of life, and physiological indicators in cancer survivors. Integr Cancer Ther 2018;17:1183–94. 30354701 10.1177/1534735418805149 PMC6247550

[12] Zigmond AS Snaith RP The hospital anxiety and depression scale. Acta Psychiatr Scand 1983;67:361–70. 6880820 10.1111/j.1600-0447.1983.tb09716.x

[13] Tung HY Chao TB Lin YH Depression, fatigue, and QoL in colorectal cancer patients during and after treatment. West J Nurs Res 2016;38:893–908. 26902798 10.1177/0193945916630256

[14] Dunstan DA Scott N Todd AK Screening for anxiety and depression: reassessing the utility of the Zung scales. BMC Psychiatry 2017;17:329. 28886698 10.1186/s12888-017-1489-6 PMC5591521

[15] Nelson CJ Cho C Berk AR Are gold standard depression measures appropriate for use in geriatric cancer patients? A systematic evaluation of self-report depression instruments used with geriatric, cancer, and geriatric cancer samples. J Clin Oncol 2010;28:348–56. 19996030 10.1200/JCO.2009.23.0201 PMC2815722

[16] Park J Shin DW Kim TH Development and Validation of the Korean Version of the European Organization for Research and Treatment of Cancer Quality of Life Questionnaire for Patients with Non-muscle Invasive Bladder Cancer: EORTC QLQ-NMIBC24. Cancer Res Treat 2018;50:40–9. 28279061 10.4143/crt.2016.594 PMC5784644

[17] Karageorge A Murphy MJ Newby JM Acceptability of an internet cognitive behavioural therapy program for people with early-stage cancer and cancer survivors with depression and/or anxiety: thematic findings from focus groups. Support Care Cancer 2017;25:2129–36. 28213818 10.1007/s00520-017-3617-8

[18] Sjamsudin E Maulina T Cipta A Assessment of oral cancer pain, anxiety, and quality of life of oral squamous cell carcinoma patients with invasive treatment procedure. Oral Maxillofac Surg 2018;22:83–90. 29332186 10.1007/s10006-018-0672-3

[19] Buscariollo DL Cronin AM Borstelmann NA Impact of pre-diagnosis depressive symptoms and health-related quality of life on treatment choice for ductal carcinoma in situ and stage I breast cancer in older women. Breast Cancer Res Treat 2019;173:709–17. 30406869 10.1007/s10549-018-5006-5

